# P02.03. Decreased medication use and cost savings in inpatient oncology through a yoga-based integrative medicine approach

**DOI:** 10.1186/1472-6882-12-S1-P59

**Published:** 2012-06-12

**Authors:** J Friedman, B Kligler, P Hommel, W Merrell

**Affiliations:** 1 Beth Israel Medical Center, Department of Integrative Medicine, New York City, USA

## Purpose

The Urban Zen (UZ) Initiative at Beth Israel Medical Center was a pilot project evaluating the impact of a multi-faceted “optimal healing environment” intervention on quality of life and cost outcomes for inpatients on a medical oncology floor. Little research exists on the question of potential cost savings. This poster presents a summary of our findings on cost outcomes from this Initiative.

## Methods

The UZ intervention included: (1) remodeling physical space; (2) holistic nursing techniques training for the nursing staff; (3) yoga therapists on the unit to work with patients using breathing and yoga techniques; (4) “patient navigator”; and (5) audiovisuals demonstrating yoga and relaxation techniques for patients to use in bed. We compared a control group admitted prior to UZ implementation to a treatment group of patients admitted after the intervention was implemented. Inclusion criteria were age 18-85, Karnofsky score ≥ 60, life expectancy ≥ 6 months, and English-speaking.

## Results

UZ group had lower mean medication costs. Total medication costs were significantly higher for the baseline group ($889) versus the UZ group ($420) for a cost savings on average of $469 per patient. Significant differences in favor of the UZ group were seen with regard to anti-nausea medications and anti-anxiety medications.

## Conclusion

A significant decrease in medication costs in the UZ group was observed compared to controls, on the order of $152 per patient. If we extrapolate this savings of approximately $50.66 per patient per day to a total of 8760 patient days per year (24 beds x 365 days), this results in a total savings to the hospital of $443,781 annually. This type of innovative, patient-centered, “optimal healing environment” intervention in the inpatient setting has the potential to significantly reduce patients’ need for medication to treat anxiety, sleep, nausea and pain. This decreased use of medications can create substantial cost savings for hospitals in the care of oncology patients.

